# Genome-wide association analysis and gene mining of flavonoids in *Xanthoceras sorbifolia*

**DOI:** 10.1038/s41598-025-00514-4

**Published:** 2025-07-01

**Authors:** Yuxue Huo, Lei Wang, Lu Lu, Dan Wu, Li Liu, Xiaoman Xie, Yongjun Zhao

**Affiliations:** 1 Key Laboratory of National Forestry and Grassland Administration on Conservation and Utilization of Warm Temperate Zone Forest and Grass Germplasm Resources, Shandong Provincial Center of Forest and Grass Germplasm Resources, Jinan, 250102 China; 2 State Key Laboratory of Tree Genetics and Breeding, National Engineering Research Center of Tree Breeding and Ecological Restoration, Key Laboratory of Genetics and Breeding in Forest Trees and Ornamental Plants, Ministry of Education, College of Biological Sciences and Biotechnology, Beijing Forestry University, Beijing, 100083 China

**Keywords:** *Xanthoceras sorbifolia*, Flavonoids, Whole genome resequencing, SNP screening, GWAS, Genetic association study, Plant breeding, Plant development, Plant hormones

## Abstract

*Xanthoceras sorbifolia* is a unique woody oilseed tree in China, and its leaves are rich in flavonoids, which are involved in plant growth, development and defense. However, the mining of flavonoid synthesis-related genes in *Xanthoceras sorbifolia* leaves is lacking. In this study, 226 leaves of *Xanthoceras sorbifolia* from eight provinces in the key distribution areas were measured for flavonoid content, and the differences in flavonoid content of *Xanthoceras sorbifolia* were analysed to screen out excellent seed sources and six excellent single plants with higher flavonoid content. Genome-wide association analysis (GWAS) was used to identify genes controlling the synthesis of flavonoids, and 62 significant Single nucleotide polymorphism (SNP) sites were identified, which were closely associated with 8 traits, and a total of 11 genes coding for proteins. We found that these genes mainly encode proteins such as WPP domain-associated protein (WAP) (Fragment), Protein pleiotropic regulatory locus 1 (PRL1) and Phosphomevalonate kinase, peroxisomal (PMK), etc. We found that these proteins may directly or indirectly affect the synthesis of flavonoids, which will provide a data base for molecular breeding and genetic improvement of *Xanthoceras sorbifolia*.

**Supplementary Information:**

The online version contains supplementary material available at 10.1038/s41598-025-00514-4.

## Introduction

*Xanthoceras sorbifolia*, also known as papaya and wenge, is a deciduous shrub or small tree of the genus *Xanthocera* in the family Sapindaceae. *Xanthoceras sorbifolia* is highly resilient and grows well in cold, dry and saline soils^1^, and is widely distributed in the northern and northeastern regions of China, such as Inner Mongolia, Liaoning and Shaanxi.

The leaves of *Xanthoceras sorbifolia* are rich in flavonoids, mainly including Myricitrin, Quercetin, Rutin, Kaempferol and L-Epicatechin. Studies have shown that flavonoids, as a widely distributed secondary metabolite in plants, are an important component of the odor and color of fruits and vegetables, and also contribute to the agronomic, industrial and nutritional value of plant products. It affects the quality of seeds and fruits, the astringency of plant products, and the health value of food products^2^. Flavonoids play an important role in the growth, development and defense of plants, and these substances have a better scavenging of free radicals so that they can resist the attack of pathogens, which is also important for human health^3^.

Flavonoids are generated from phenylalanine through the phenylpropanoid pathway^4^. In this pathway, phenylalanine, an aromatic amino acid, is converted to p-coumaroyl-CoA through the activity of phenylalanine ammonia lyase (PAL), cinnamic acid 4-hydroxylase (C4H), and 4-coumarate: CoA ligase (4 CL). PAL catalyzes the first committed step in the general phenylpropanoid pathway, namely, the deamination of phenylalanine to trans-cinnamic acid^5^. The second step in the general phenylpropanoid pathway involves the activity of C4H, a cytochrome P450 monooxy-genase in plants, which catalyzes the hydroxylation of trans-cinnamic acid to generate p-coumaric acid. This is also the first oxidation reaction in the flavonoid synthesis pathway^6^. In the third step of the general phenylpropanoid pathway, 4 CL catalyzes the formation of p-coumaroyl-CoA by the addition of a co-enzyme A (CoA) unit to p-coumaric acid. PAL and 4 CL are encoded by gene families with multiple members, e.g., up to 20 members of the *Solanum tuberosum* PAL gene family^7^. In plants, the activity of 4 CL is positively correlated with the anthocyanin and flavonol content in response to stress^8^, while PAL, C4H, and 4 CL are often coordinately expressed^9^. Chalcone synthase (CHS) is the first key enzyme in the next process, leading the phenylpropanoid pathway to flavonoid synthesis. This enzyme catalyzes the reaction between p-coumaroyl-CoA and malonyl-CoA to synthesize chalcone^10^. CHS is one of the most abundant enzymes in the phenylpropanoid pathway, but its catalytic efficiency is low. The transcription of CHS in plants was inhibited by high concentrations of cinnamic acid and promoted by high concentrations of coumaric acid^11^. The key enzyme in the next step is chalcone isomerase (CHI), which catalyzes the further synthesis of chalcone into flavanone, the precursor of isoflavone, and thus enters the isoflavone metabolic branch^10^. Naringenin is a flavanone. In the analysis of the genes related to the synthesis of flavonoids in Trollius chinensis, Wang et al. found that chalcone was isomerized to naringenin under the catalytic effect of CHI, and naringenin, as a major metabolite, could be generated under the catalytic effect of different enzymes to produce a variety of products, which could be generated under the effect of flavanone-3-hydroxylase (F3H) and flavonol synthase (FLS) to produce Kaempferol, and Kaempferol could be generated under the catalytic effect of flavonoid 3’,5’-hydroxylase (F3’5’H) to produce Quercetin, and so on^12^.

Whole genome resequencing has been widely used in animal, plant and microbial fields^13^, and with the reduction of the cost of second-generation sequencing technology, the scale of whole genome resequencing in plants has been expanding, and 187 species of plants have been reported to have been subjected to whole genome resequencing^14^, and these large-scale whole-genome resequencing has promoted the construction of a complete map, and accelerated the improvement of crops. GWAS based on whole-genome resequencing is a technique that uses variation among individuals in a target group to determine molecular markers associated with complex trait variation, and then unearths genes related to the target traits, and has been widely used in various fields. Xia et al.^15^ determined the total flavonoid content in brown rice using core rice germplasm containing 633 copies from 32 countries, and a total of 53 quantitative trait loci (QTLs) were detected and eight candidate genes were identified through a genome-wide association study. Zhao^16^ investigated 208 *Xanthoceras sorbifolia* germplasm resources for important agronomic traits, and explored SNP loci and candidate genes associated with important agronomic traits such as leaf water content and leaf hairiness through GWAS, and screened out transcription factors related to the target traits, mainly bHLH85 and R2R3-MYB, in addition to a number of genetically encoded proteins.

There is a lack of research on the synthesis pathways of flavonoids and their related gene mining in *Xanthoceras sorbifolia* plants. In order to promote the basic research and comprehensive development and utilisation of *Xanthoceras sorbifolia*, and to promote its better growth and development, so that people can make fuller use of the value of *Xanthoceras sorbifolia*, the present study, through further isolation and analysis, identified the differences in flavonoid content of *Xanthoceras sorbifolia* leaves from different seed sources, and used this characteristic law to select the excellent single plants with higher flavonoid quality, so as to provide the basis for the development of *Xanthoceras sorbifolia* related to the flavonoids of the new varieties. Whole genome resequencing and genome-wide association analyses will be used to explore the genetic factors affecting the flavonoid content of *Xanthoceras sorbifolia* and the molecular mechanism of its synthesis, so as to provide a basis for molecular breeding and genetic improvement of *Xanthoceras sorbifolia*.

## Materials and methods

### Plant materials

**Fig. 1:**
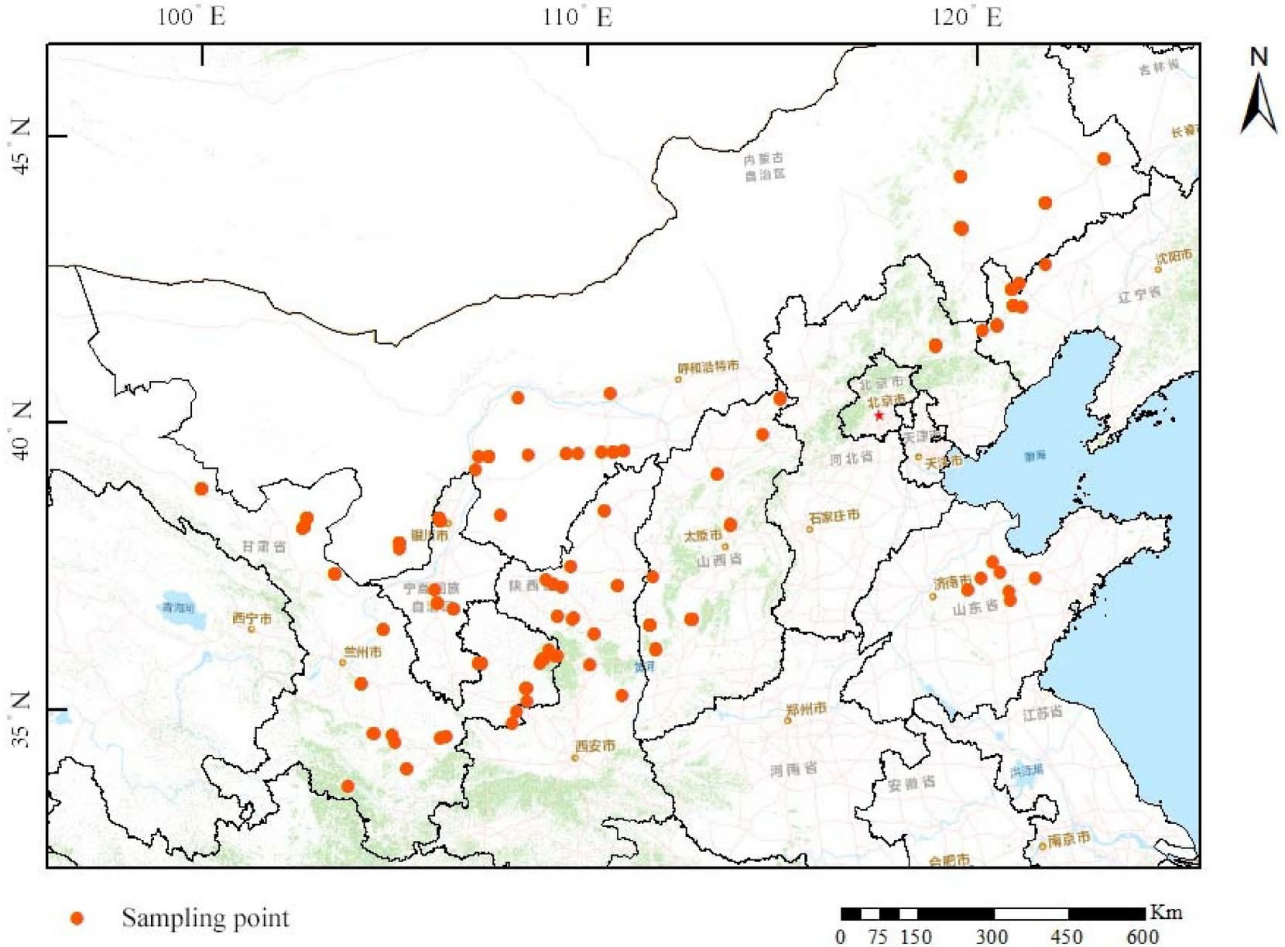
Distribution map of seed source areas for 226 *Xanthoceras sorbifolia* materials. Mapping with ArcGIS (v10.8, https://www.esri.com/zh-cn/arcgis/).

In 2021, 226 seeds of *Xanthoceras sorbifolia* were collected from eight provinces and sown in Zaoyuan Conservation Bank of the Shandong Provincial Center of Forest and Grass Germplasm Resources, Jinan, Shandong Province. *Xanthoceras sorbifolia* resource centre is managed with regular watering and irrigation. Leaf collection was carried out in May 2023, and the plants averaged 0.58 m in height, with sturdy branches, lush foliage, and good growth potential. For each material, well-grown *Xanthoceras sorbifolia* seedlings were randomly selected, and leaves were collected from the upper, middle, and lower parts of the plant, as well as from the outer and inner parts of the crown, using good growth conditions and the absence of pests and diseases as the criteria for leaf collection. The samples were rapidly placed in liquid nitrogen for cryogenic transport after collection, and stored in a −80 °C refrigerator for backup. Information on the distribution of sampling locations is shown in Fig. 1.

### Determination of flavonoid content

Take the standard and dissolve it with methanol to make 5000 ng/ml standard stock solution. Take a certain volume of configured standard reserve solution, methanol dilution to different concentrations and then mixed to obtain different concentrations of mixed standards. The leaves of *Xanthoceras sorbifolia* were rapidly ground with liquid nitrogen, 0.5 g of the ground powder was taken in 3 repetitions, weighed precisely and dissolved in 75% ethanol solution by volume. Extraction was performed with an ultrasonic cleaner (250 W, room temperature 25 °C) for 20 min and centrifuged (20 min, 10000 rpm, 4 °C). Dilute 5 times. Take 1 ml of the diluted solution and pass it through a 0.22 μm microporous organic filter membrane for on-line detection.

A Thermo Fisher UltiMate 3000 liquid phase system was used, and the chromatographic column: Agela Technologies-Innoval ODS-2-C_18_ column (2.1 mm × 75 mm, 5 μm); Mobile phase A: methanol, mobile phase B: water (containing 0.1% formic acid by volume)^17,18^. Gradient elution with the following gradient procedure: 0 ~ 2 min: 97% B ~ 97% B, 2 ~ 4 min: 97% B ~ 95% B, 4 ~ 6 min, 95% B ~ 80% B, 6 ~ 9 min: 80% B ~ 50% B, 9 ~ 11 min: 50% B ~ 40% B, 11 ~ 13 min: 40% B ~ 5% B, 13 ~ 20 min: 5% B ~ 5% B, 20 ~ 20.1 min: 5% B ~ 60% B, 20.1 ~ 22 min: 60% B ~ 97% B, 22 ~ 25 min: 97% B ~ 97% B; Column temperature: 40 °C, injection volume: 5 µL, equilibrated for 10 min before injection, flow rate: 0.3 ml/min. An AB Sciex Triple Quad 3200 mass spectrometry detection system was used. The ion source is an electrospray ion (ESI) source, negative ion scanning mode; inlet voltage (EP): −10 V; ion spray voltage: −4500 V. Nitrogen was passed through the entire process, with each gas pressure set as follows, curtain gas: 30 psi; atomising gas (Gas 1): 50 psi; and heating gas (Gas 2): 55 psi. Interface heater: open state; turbo spray temperature: 550 °C. In the present study, multiple reaction monitoring (MRM) mode was used for the quantification of flavonoids in the leaves of *Xanthoceras sorbifolia*. The monitored ion pairs^19^ and other parameters of the tested components are shown in Table 1. The peak times of the standards and samples were the same for each compound, the parameters of the method were set reasonably, and the compounds identified were the target compounds (Fig. S1, S2).

**Table 1:** Mass spectrometry detection parameters of seven flavonoids of *Xanthoceras sorbifolia* leaves.

Compounds	Retention time (min)	Q1 Mass	Q3 Mass	Scanning time (msec)	DP (V)	CE (V)
Rutin	12.01	609.1	300.0	70	−84	−53
Myricitrin	11.81	462.9	316.0	70	−68	−35
(−)-Epigallocatechin	0.82	304.9	125.1	70	−42	−28
L-Epicatechin	9.77	288.9	108.8	70	−46	−35
Quercetin	12.89	300.7	150.9	70	−56	−30
(+)-Gallocatechin	0.82	304.8	124.9	70	−52	−29
Kaempferol	13.54	284.7	93.1	70	−70	−50

Note: Q1 Mass: mass setting of the first quadrupole; Q3 Mass: mass setting of the third quadrupole; DP: declustering potential; CE: collision energy.

### Whole genome resequencing

In this study, the Illumina platform was used for sequencing, and the QC-qualified libraries were up-sequenced with a fragment size of 150 bp bipartite sequence. The raw image data files were obtained and converted into sequenced reads by CASAVA Base Calling analysis. Raw Data is filtered for artificial joint sequences and low-quality sequences. Specifically, splice-contaminated reads (reads with splice-contaminated bases greater than 5 bp), low-quality reads (more than 50% of reads with base quality values below 19), and reads with an N-content ratio greater than 5% are removed.

### Population variation detection

The superior strain of *Xanthoceras sorbifolia* “WF18”^20^ was used as the reference genome, and the filtered Clean reads were compared to the reference genome using genome comparison software Burrows-Wheeler Aligner (BWA, v0.7.9a, https://bio-bwa.sourceforge.net/)^21^ in mem mode. The sequences were sequenced after comparison using Samtools software (v0.1.19, https://samtools.sourceforge.net/)^22^ and low quality (MQ < 4) reads were removed, after which PCR repeats were removed by Picard-tools (v1.13, http://broadinstitute.github.io/picard/) software.

SNP sites and Insertion and Deletion (InDel) sites were detected using the mutation analysis software Genome Analysis Toolkit (GATK, v3.3-0, http://www.broadinstitute.org/gsa/wiki/index.php/The_Genome_Analysis_Toolkit)^23^ using Haplotype Caller mode and joint calling methods. Further filtering and screening was done based on quality value, depth and repeatability (SNP: QD < 2.0, ReadPosRankSum < −8.0, FS > 60.0, QUAL < 30.0, DP < 4.0, MQ < 40.0, MappingQualityRankSum < −12.5; InDel: QD < 2.0, ReadPosRankSum < −20.0, FS > 200.0, QUAL < 30.0, DP < 4.0), and the detected variant sites were annotated accordingly using ANNOVAR software (v20160423, http://www.openbioinformatics.org/annovar/)^24^ and the resulting genome annotation files (gff/gtf) of *Xanthoceras sorbifolia*.

The vcf files of SNPs were converted to ped format by VCFtools software (v0.1.15, https://vcftools.github.io/index.html)^25^ or the ped files were filtered directly to retain only the valid loci on the main chromosome (removing contig as well as scaffold). Filtering was performed using PLINK software (v1.9, https://www.cog-genomics.org/plink/1.9/)^26^, loci with a minimum allele frequency of less than 0.05 were removed, loci and individuals with deletion rates greater than 10% were removed, and loci with Hardy-Weinberg equilibrium less than 10E-6 were removed. The number of valid SNPs was also calculated by the Genetic Type I error calculator software (GEC, v0.2, http://statgenpro.psychiatry.hku.hk/gec/)^27^ to obtain the Significant P Value value, which was used as a threshold for subsequent GWAS results after Bonferroni correction.

### Population genetic analysis

Population structure analysis was carried out using Admixture (v1.3.0, http://www.genetics.ucla.edu/software)^28^, with K taking the values of 2 ~ 10, and the K value with the smallest Cross-Validation (CV) error was taken as the optimal K value according to the CV cross validation method. According to the Neighbour-Joining (NJ) algorithm, the evolutionary relationships between the samples were calculated using the PHYLogeny Inference Package software (Phylip, v3.696, https://phylipweb.github.io/phylip/)^29^ and the resultant graphs of the evolutionary trees were plotted using Newick utils (v1.6, http://cegg.unige.ch/newick_utils)^30^. The linkage disequilibrium (LD) was obtained by calculating r^2^ by the software PopLDdecay (v1.29, https://github.com/BGI-shenzhen/PopLDdecay)^31^, while the distance corresponding to half of the maximum value of r^2^ was taken as the half-loss distance.

### GWAS

In this study, Genome-wide Efficient Mixed Model Association software (GEMMA, v0.94, http://stephenslab.uchicago.edu/software.html)^32^ was used for association analysis using linear mixed model (LMM). The results were corrected for multiple testing using the Bonferroni method, and compared with the threshold value (Significant P Value) calculated by the GEC v0.2 software, if the specified threshold line was exceeded it was used as a candidate locus associated with the target gene, if the threshold line was not exceeded the top 10 loci with the smallest arrangement of p-values were used as candidate loci. A range of 50000 bp upstream and downstream of the candidate site was selected as the candidate interval. Manhattan and Quantile-quantile (QQ) plots were drawn with R (v3.3.1, https://cran-archive.r-project.org/bin/windows/base/old/3.3.1/) to demonstrate the results of GWAS. Functional annotation of the genes in the candidate region was carried out, and the mRNA sequences of the genes were extracted from the fa and gff files, and then the sequences were blasted with the Uniprot database, and the optimal comparison results with an e value of less than 1e^−5^ were selected to extract the corresponding annotation information in the database.

## Results

### Analysis of variance of flavonoid content in *Xanthoceras sorbifolia*

The results of ANOVA of flavonoid composition of different groups of *Xanthoceras sorbifolia* are shown in Table 2, except for Myricitrin, L-Epicatechin and Kaempferol, there were highly significant and significant differences in other indexes, indicating that the content of most of the flavonoids in *Xanthoceras sorbifolia* leaves was widely varied and the diversity was high.

**Table 2:** Analysis of variance of flavonoid components contained in the leaves of *Xanthoceras sorbifolia* from different populations.

Character/Index	Mean square		F value
	Intergroup	In-group	
Rutin	47259350.16	8000518.30	5.91**
Myricitrin	21116894.18	21349618.35	0.99
(−)-Epigallocatechin	226531.37	87309.62	2.60*
L-Epicatechin	4732653.65	4514641.71	1.05
Quercetin	1714250.00	103010.10	16.64**
(+)-Gallocatechin	287583.86	107394.37	2.68*
Kaempferol	9660.93	4921.37	1.96
Total flavonoids	4570597712.23	378408506.85	12.08**

‘**’ indicates highly significant differences (*p* ≤ 0.01) and ‘*’ indicates significant differences (*p* ≤ 0.05).

The results of intergroup analysis of flavonoid content in different groups of *Xanthoceras sorbifolia* are shown in Table 3, and the contents of seven components of flavonoids and total flavonoids in *Xanthoceras sorbifolia* leaves showed a certain range of variability among different individuals. The range of variation of L-Epicatechin was the largest, with the maximum value of 15726.89 mg/kg for the plants in Gansu, the minimum value of 41.17 mg/kg for the plants in Inner Mongolia, the maximum value was about 382 times of the minimum value, and the mean value was 1649.82 mg/kg. Myricitrin had the smallest range of variation from 7954.35 mg/kg (Inner Mongolia) to 50275.6 mg/kg (Inner Mongolia), and the maximum value was 6.32 times of the minimum value. Rutin, Quercetin, Kaempferol and total flavonoids also showed some range of variation, but the coefficients of variation were lower compared to L-Epicatechin. Among the seven flavonoids, Rutin and Myricitrin had the largest mean values, and both of them were more abundant in the leaves of *Xanthoceras sorbifolia* than other flavonoids.

**Table 3:** Inter-population variation analysis of flavonoid content in leaves of *Xanthoceras sorbifolia* from different populations.

Character/Index	Average value	Standard deviation	Coefficient of variation	Minimum value	Maximume value
Rutin	7345.44	3036.76	0.41	699.45	20440.13
Myricitrin	16617.05	4619.78	0.28	7954.35	50275.60
(−)-Epigallocatechin	301.97	302.72	1.00	3.32	2447.81
L-Epicatechin	1649.82	2126.36	1.29	41.17	15726.89
Quercetin	1078.31	391.33	0.36	399.84	2825.26
(+)-Gallocatechin	338.46	336.16	0.99	5.44	2542.92
Kaempferol	171.81	71.20	0.41	54.67	470.76
Total flavonoids	57255.59	22557.31	0.39	16404.00	120135.00

### Principal component analysis of flavonoid content in *Xanthoceras sorbifolia*

The principal component analyses of seven flavonoid components and total flavonoids content of different seed sources were shown in Table 4, and the eigenvalues of the four principal components were all greater than 0.7, with their cumulative contribution rate of 85.47%, so the flavonoid quality of *Xanthoceras sorbifolia* leaves could be judged according to the four principal components.

**Table 4:** Principal component analysis of flavonoid constituents of *Xanthoceras sorbifolia* leaves.

Character/Index	Loadings for each principal component			
	PCA 1	PCA 2	PCA 3	PCA 4
(+)-Gallocatechin	0.91	0.25	−0.14	−0.25
(−)-Epigallocatechin	0.91	0.25	−0.12	−0.27
Total flavonoids	0.73	−0.18	0.23	0.45
Myricitrin	0.12	0.82	0.33	−0.25
Kaempferol	−0.29	0.74	0.04	0.44
Quercetin	−0.47	0.73	−0.05	−0.02
L-Epicatechin	0.46	0.25	−0.71	0.36
Rutin	0.56	0.03	0.61	0.24
Eigenvalue	3.02	1.96	1.08	0.78
Cumulative contribution rate	37.73%	62.21%	75.69%	85.47%

Calculating the composite scores of the principal components can screen out the good single plants with high quality of flavonoids in *Xanthoceras sorbifolia* leaves, and can evaluate the quality of flavonoids in *Xanthoceras sorbifolia* leaves from different sources, the higher the composite score, the better the quality of flavonoids in *Xanthoceras sorbifolia* leaves. The top 10 single plants ranked by the comprehensive score were screened as shown in Table 5, which had high flavonoid quality, and the total flavonoid content of the Inner Mongolia group ZYJ030, Shaanxi group 2021ZYJ004, Gansu group 2021 WL088, Shanxi group 2021 WD032, Inner Mongolia group 2021 WL034, and Gansu group 2021 WL065 plants was more than 100000 mg/kg, and these materials can be used as good single plants with high flavonoid content for the selection of good varieties. The comprehensive evaluation of flavonoid quality of *Xanthoceras sorbifolia* leaves from different seed sources is shown in Table 6. It was found that the flavonoid quality of *Xanthoceras sorbifolia* leaves from Gansu seed source area was higher, with an average total flavonoid content of 71655.75 mg/kg, while the other seed sources were in the order of Shanxi (67810.62 mg/kg), Ningxia (65235.65 mg/kg), Shaanxi (62710.42 mg/kg), Shandong (59650.85 mg/kg), Inner Mongolia (44186.68 mg/kg), Liaoning (41283.00 mg/kg), Hebei (32809.33 mg/kg).

**Table 5:** Excellent single plants regarding the flavonoid composition of *Xanthoceras sorbifolia* leaves obtained by calculating the composite score of the principal component analysis.

Source	Monoculture	Principal component score					Overall ranking
		PCA 1	PCA 2	PCA 3	PCA 4	Aggregate score	
Inner Mongolia	ZYJ030	56814.88	−2892.77	33745.08	59693.99	28385.34	1
Shaanxi	2021ZYJ004	53678.53	−4300.67	33313.01	57484.73	26972.14	2
Gansu	2021 WL088	53323.18	−1025.59	32591.81	55138.27	26584.36	3
Shanxi	2021 WD032	48960.62	−1888.26	27393.29	52521.7	24511.31	4
Gansu	2021 WL065	48630.45	−1699.67	19145.88	53858.23	24245.04	5
Inner Mongolia	2021 WL034	48220.1	−1396.39	31719.8	49880.16	24116.37	6
Inner Mongolia	2021 WL029	49910.34	12205.26	38567.36	40750.27	24085.34	7
Inner Mongolia	2021 WL037	46333.81	−1652.43	29844.99	48089.5	23167.74	8
Ningxia	2021 WL016	45079.54	394.77	31413.29	46333.75	22574.69	9
Shaanxi	2021ZYJ023	45211.82	213.71	28259.41	45852.07	22473.04	10

**Table 6:** Ranking of species origin regarding the flavonoid constituents of *Xanthoceras sorbifolia* leaves obtained by calculating the composite score of the principal component analysis.

Source	Principal component score					Overall ranking
	PCA 1	PCA 2	PCA 3	PCA 4	Aggregate score	
Gansu	33368.80	2177.57	24184.94	32666.51	16581.27	1
Shanxi	32916.17	1635.95	23293.51	32700.52	16384.48	2
Ningxia	31498.38	2158.40	23582.73	30873.40	15680.48	3
Shaanxi	30537.46	2760.69	22473.24	29678.49	15164.42	4
Shandong	27652.67	4959.09	20168.65	25689.69	13609.72	5
Inner Mongolia	22052.65	5784.41	17821.52	19587.45	10822.87	6
Liaoning	20899.48	6813.75	17470.61	18035.40	10224.41	7
Hebei	18453.61	6401.02	16078.26	15515.82	9009.35	8

### Whole genome resequencing and population variation detection

**Fig. 2:**
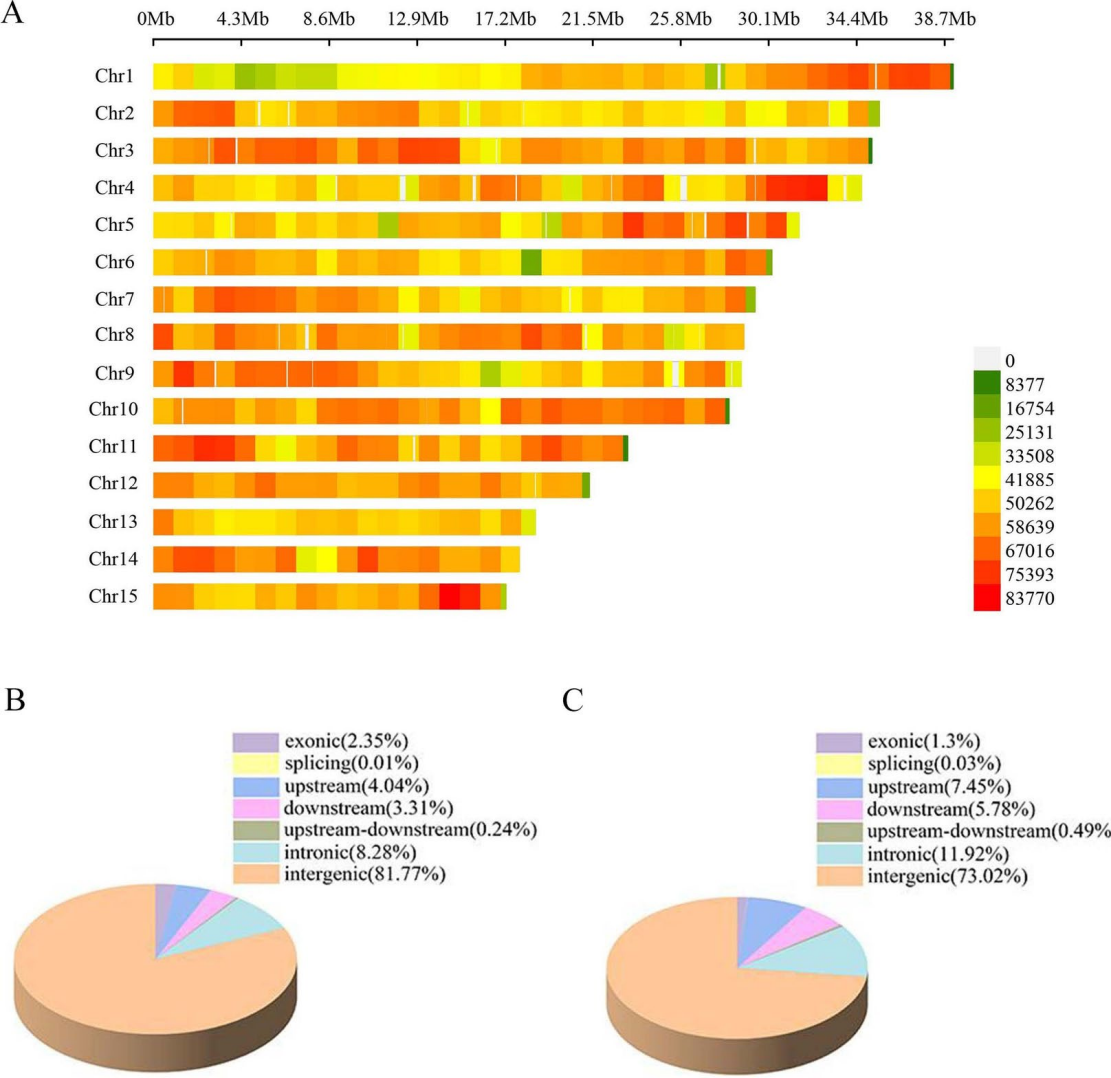
(**A**) SNP density distribution. The abscissa is the length of the chromosome. Each band represents one chromosome; (**B**) Pie chart of SNP position distribution; (**C**) Pie chart of InDel position distribution.

High-throughput sequencing was performed on 104 samples of *Xanthoceras sorbifolia*, resulting in 255 Gb of raw data. After filtering, 250 Gb of clean data was obtained. The quality of library construction was better and the sequencing quality was relatively high, with an average sequencing depth of 12.4 (Fig. S3). The comparison rate to the reference gene averaged 90.8% (Fig. S4), which is a high comparison rate. A total of 23540497 population SNPs and 3984005 InDel sites were detected after the comparison (Fig. 2A), and the SNP sites mainly occurred in the intergenic region, accounting for 81.77% (Fig. 2B). The SNPs were subjected to quality control (Fig. S5), and 4556824 SNPs were obtained after quality control (Tab. S1), and 2329521.8 SNPs that could be used for GWAS were finally obtained (Tab. S2).

### Group structure analysis

**Fig. 3:**
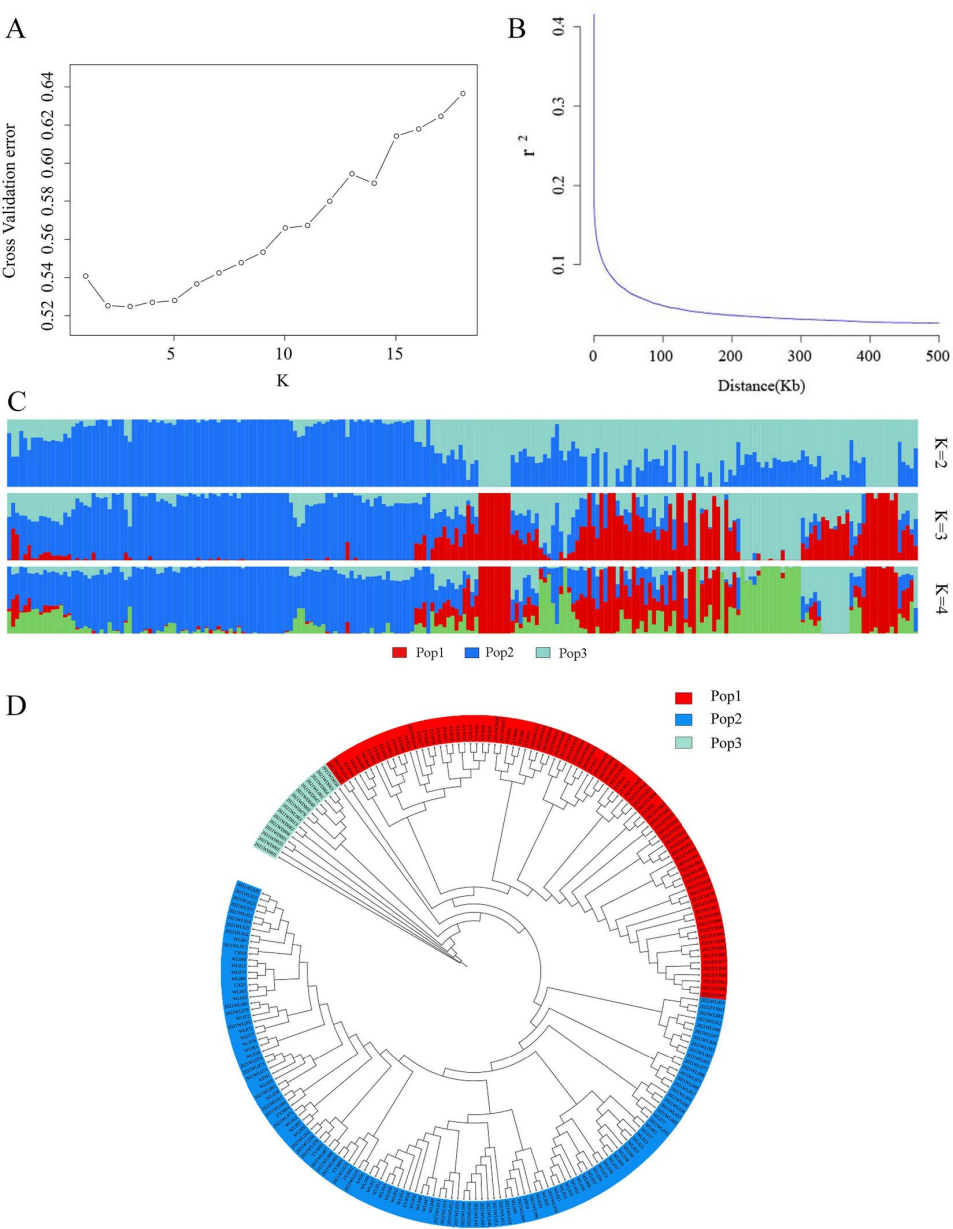
Group Structure of 226 *Xanthoceras sorbifolia* samples. (**A**) Diagram showing the value of 226 samples based on clustering from 1 to 18, X-axis is K value 1–18, Y-axis is cross-validation errors; (**B**) LD decay plot. The LD decay distance is defined as the distance at which the LD coefficient r^2^ reaches half of its maximum value; (**C**) Clustering analysis when the number of subgroups is in the range 2–4, the colours represent separate groups; (**D**) Phylogenetic tree of 226 *Xanthoceras sorbifolia* samples.

Due to the non-random distribution of genetic variation in a population, which leads to the division of the whole population into specific subgroups, the dynamic pattern of change in population differentiation due to genetic variation in population evolution can be demonstrated by the structure of the population, which determines the subgroup to which an individual belongs. The results of this study showed that the CV error was the smallest when K = 3 (Fig. 3A), so it was most appropriate to divide this *Xanthoceras sorbifolia* population into three subgroups.

As shown in Fig. 3C, the K = 2, K = 3, and K = 4 results are displayed to show the proportion of genetic background of each subpopulation that each individual has, and the three subpopulations into which they are divided are labelled as subpopulation 1, subpopulation 2, and subpopulation 3 based on different ancestries in the K = 3 stacked plot, which are indicated in red, blue, and cyan, respectively. The fact that the various source plants are distributed in different subpopulations suggests that the differences brought about by the geography of the seed source do not create a degree of geographic isolation, and that there is genetic exchange between the regions.

### Phylogenetic tree construction

The evolutionary tree was constructed based on the proximity of kinship between different samples, and the filtered SNPs were used to construct Fig. 3D using the NJ method using PHYLIP v3.696 software. The more closely related samples were pooled together and divided into three branches, which were marked using different colours to form three subgroups, which was in good agreement with the population structure and the results of the principal component analysis.

### Analysis of LD results

As shown in Fig. 3B, r^2^ decays from 0.44 to 0.026, and the distance is 100 bp when r^2^ falls to half of its maximum value (0.22), so the population has a decay distance of 100 bp. A larger LD decay distance indicates a slower rate of decay, suggesting that the population has been subject to selection and that the population is less genetically diverse. The results showed that when r^2^ decreased to 0.2, the distance was 200 bp; when r^2^ decreased to 0.15, the distance was 1.7 kb; and when r^2^ decreased to 0.1, the distance was 14.5 kb, so the LD decayed faster and the genetic diversity of this population was higher.

### Results of GWAS

**Fig. 4:**
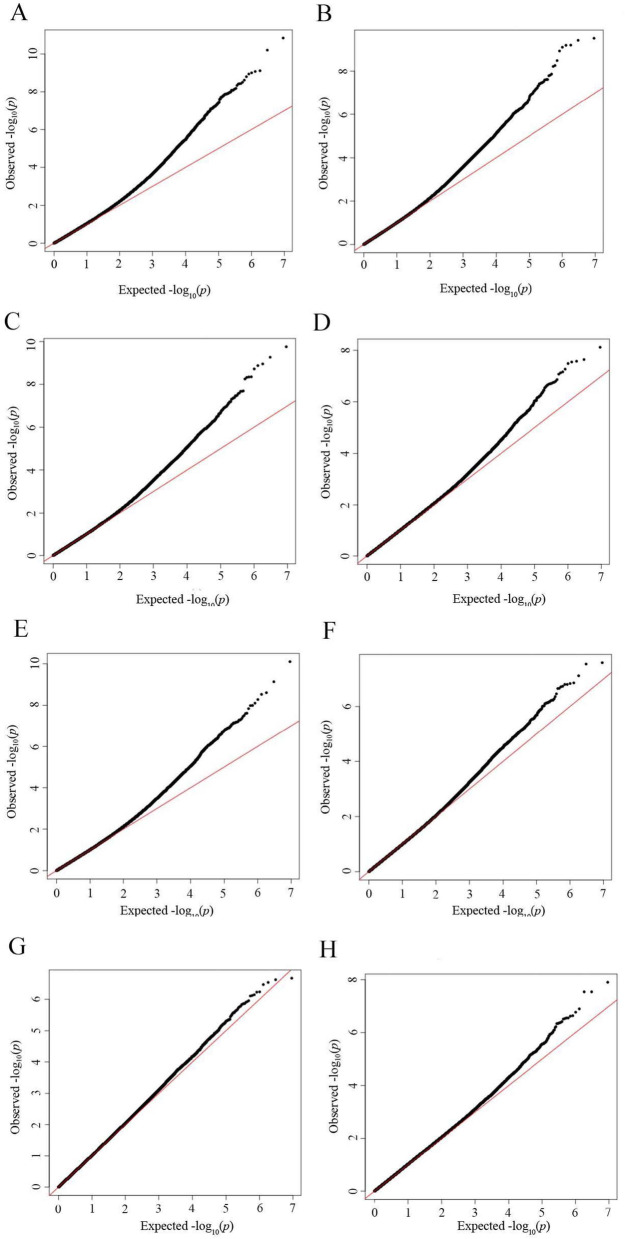
Quantile-quantile (Q-Q) plots illustrating the distribution of observed p-values compared to expected p-values for flavonoids in *Xanthoceras sorbifolia* leaves. (**A**) L-Epicatechin; (**B**) (−)-Epigalocatechin; (**C**) (+)-Gallocatechin; (**D**) Kaempferol; (**E**) Myricitrin; (**F**) Quercetin; (**G**) Rutin; (**H**) Total flavonoids.

**Fig. 5:**
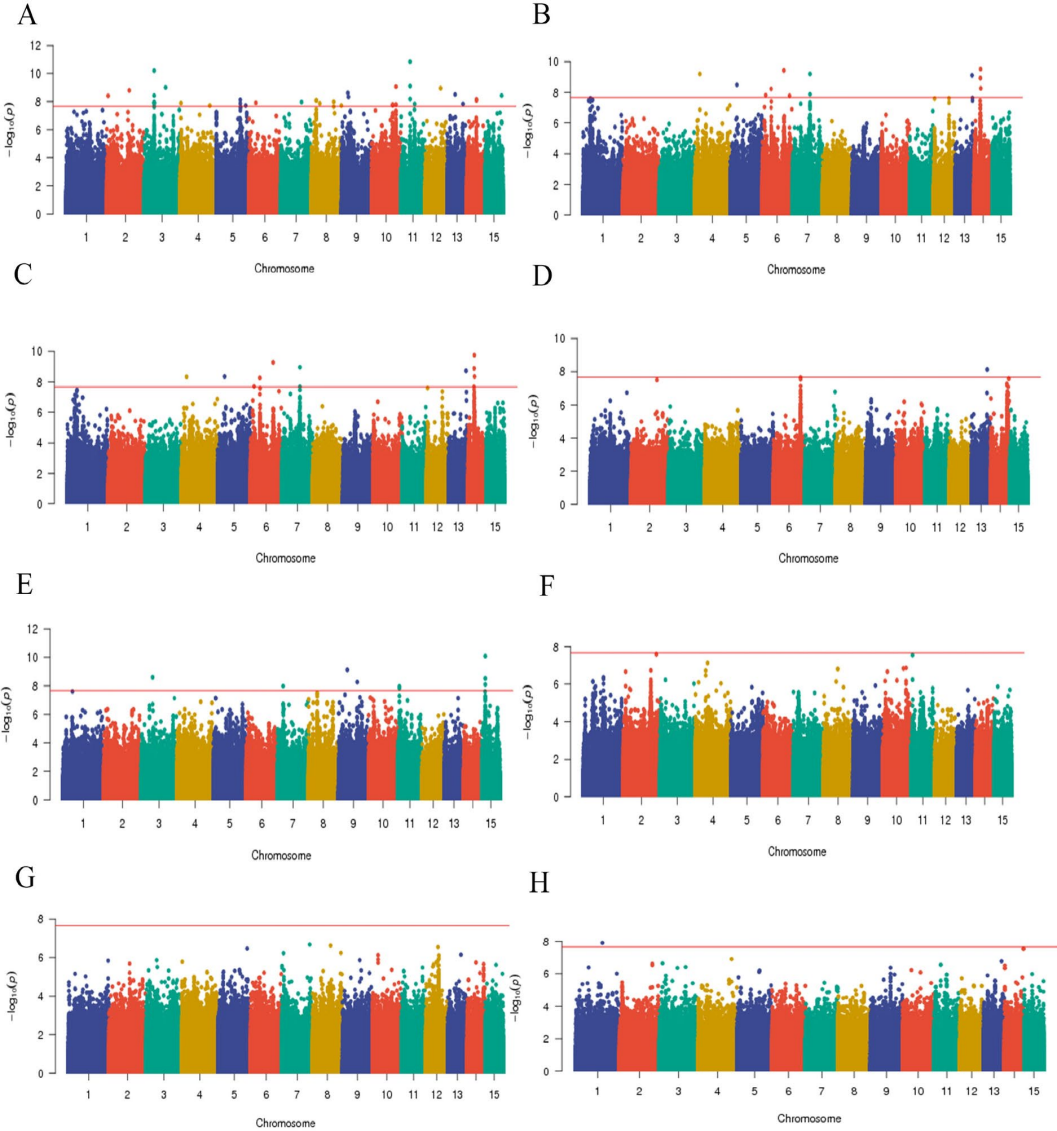
Manhattan plots of association results of LMM model for flavonoid content traits, the X-axis indicates the SNPs along each chromosome, the Y-axis is the -log 10 for the association, the horizontal straight line indicates the threshold of significant association. Each dot represents a SNP. (**A**) L-Epicatechin; (**B**) (−)-Epigallocatechin; (**C**) (+)-Gallocatechin; (**D**) Kaempferol; (**E**) Myricitrin; (**F**) Quercetin; (**G**) Rutin; (**H**) Total flavonoids.

The L-Epicatechin content was correlated with the SNP locus. As shown in Fig. 4A, the tail end of the QQ plot is warped, indicating that a locus associated with L-Epicatechin content exists and that the data are relatively reliable. As shown in Fig. 5 A Manhattan plot, there were 39 SNPs associated with L-Epicatechin content, two SNPs were found on chromosome 2, nine on chromosome 3, two on chromosome 4, three on chromosome 5, one on chromosome 6, one on chromosome 7, five on chromosome 8, two on chromosome 9, four on chromosome 10, four on chromosome 11, one on chromosome 12, two on chromosome 13, two on chromosome 14, and one on chromosome 15. Associating the SNPs with (−)-Epigallocatechin, the QQ and Manhattan diagrams are shown in Figs. 4B and 5B, respectively, there are 12 SNPs associated with (−)-Epigallocatechin, of which 1 was screened located on chromosome 4, 1 was screened located on chromosome 5, 4 were screened on chromosome 6, 7 were screened on chromosome 2, 1 on chromosome 13 and 3 on chromosome 14. Associating the SNPs with (+)-Gallocatechin, the QQ and Manhattan diagrams are shown in Figs. 4C and 5C, respectively, with 12 SNPs associated with the (+)-Gallocatechin, one on chromosome 4, one on chromosome 5, three on chromosome 6, two on chromosome 7, one on chromosome 13, and four on chromosome 14. Associating the SNPs with Kaempferol, the QQ and Manhattan diagrams are shown in Figs. 4D and 5D, respectively. There is only one locus associated with Kaempferol, located at Chr13:15192478, with a p-value of 7.57E-9. Associating SNPs with the Myricitrin, the QQ and Manhattan diagrams are shown in Figs. 4E and 5E, respectively. There are nine SNPs associated with Myricitrin, and one SNP locus was screened on chromosome 3, one on chromosome 7, two on chromosome 9, three on chromosome 15, and two on chromosome 11, respectively. Associating SNPs with Quercetin, the QQ and Manhattan diagrams are shown in Figs. 4F and 5F, respectively, under the significance threshold condition of a = 0.05, no SNP associated with Quercetin were found. Associating SNPs with Rutin, the QQ and Manhattan diagrams are shown in Figs. 4G and 5G, respectively, under the significance threshold condition of a = 0.05, no SNP associated with Rutin was found, but the 10 SNPs with the smallest p-value were selected as candidate SNP. Correlating the SNPs with Total flavonoids, the QQ and Manhattan diagrams are shown in Figs. 4H and 5H, respectively, one SNP associated with Total flavonoids content existed, located at Chr1:23886516.

## Discussion

This study showed that the flavonoid qualities of plants from eight seed source locations were in the following order from high to low: Gansu, Shanxi, Ningxia, Shaanxi, Shandong, Inner Mongolia, Liaoning, and Hebei, and that the flavonoid content increased sequentially from east to west in terms of the geographic location of the seed sources. The general trend of annual precipitation distribution from east to west in China is decreasing from south-east to north-west, and the area with the strongest light is the Qinghai-Tibet Plateau, with the lowest in the Sichuan Basin, and the radiation and light gradually increase from east to west. Some studies have shown that altitude and Ultraviolet radiation b (UV-B) radiation affect the flavonoid content^33–35^, for example, a positive correlation between Ultraviolet (UV) and Quercetin concentration in *Betula platyphylla*^36^, and a positive correlation between Quercetin and Luteolin concentration and UV intensity in *Ligustrum lucidum* leaves^37^. Azuma et al.^38^ showed that temperature and light could regulate the synthesis of flavonoids in grape berry skin by modulating the expression of three MYB-related genes (*VlMYBA1-3*, *VlMYBA1-2* and *VlMYBA2*) and other genes in the flavonoid synthesis pathway. Lv et al.^39^ showed that the contents of flavonoids, and the expression levels of genes involved in flavonoid biosynthesis (*PAL*, *C4H*, *4 CL*, *CHS1* and *DTX41*) were enhanced in response to UV-B compared to CK. Wang et al.^40^ studied the effect of climatic conditions on flavonoid content in *Fagopyrum tataricum*, light intensity can promote the accumulation of flavonoids and the expression of key enzyme genes, and the expression levels of PAL and 4 CL were positively correlated with the flavonoid content of *Fagopyrum tataricum*. This suggests that the high flavonoid content in the leaves of the plants from the Gansu seed source in this study may be due to the suitable environmental factors in Gansu that promoted the expression of genes related to flavonoid synthesis, which in turn increased the flavonoid content. In addition, there were associations between changes in drought conditions^41,42^, rainfall and temperature^43^ and flavonoid content, with drought-resistant plants up-regulating relevant structural genes and promoting flavonoid biosynthesis in response to the negative effects of water deficit conditions the drier the environment, while non-drought-resistant plants were found to have lower flavonoid content the more they were deprived of water^44,45^. However, *Xanthoceras sorbifolia* is a typically drought-resistant plant, suggesting that less precipitation may promote the accumulation of flavonoid content within *Xanthoceras sorbifolia* plants. Among the materials of this study, Inner Mongolia plant ZYJ030, Shaanxi plant 2021ZYJ004, Gansu plant 2021 WL088, Shanxi plant 2021 WD032, Inner Mongolia plant 2021 WL034 and Gansu plant 2021 WL065 scored high composite scores. The leaves had the best flavonoid quality and had a total flavonoid content of more than 100,000 mg/kg. These materials can be used as target plants with high flavonoid content for selection of superior varieties.

Candidate intervals were identified by the selected significant sites, genes present within the intervals were searched for, and these genes were annotated separately, and a total of 11 protein-coding genes were located. The analyses revealed the identification of six genes that may be involved in flavonoid synthesis. There are four genes related to L-Epicatechin, *evm.TU.Chr12.585*, *evm.TU.Chr2.1108*, *evm.TU.Chr9.385*, *evm.TU.Chr9.420*. The main proteins encoded by genes with SNP loci closely associated with L-Epicatechin are WAP (Fragment), PRL1, PMK, and Phenylacetaldehyde reductase (PME53). The WAP is located on the Golgi apparatus, and it has been found that in tomato it binds specifically to MAF1 via a convoluted helical structural domain^46^, which binds to and inhibits RNA polymerase (pol) III, thereby inhibiting the synthesis of tRNAs and other non-coding RNAs^47^. However, Sharma et al.^48^ reported that a miRNA (miR858) primary product in *Arabidopsis thaliana* encoding the peptide priPEP858a can regulate the process of flavonoid biosynthesis. Elevated miR858a gene expression levels and reduced transcript levels of the corresponding target genes are accompanied by reduced levels of total anthocyanins and flavonoid hormones, which in turn affects important functions in plant development, thus WAP may have some indirect effects on flavonoid synthesis. Flores-Pérez et al.^49^ showed that PRL1 regulates sugar-responsive and hormone metabolism in *Arabidopsis thaliana* through inhibition of SNF1-associated protein kinase, interacts with ATHKAP2, an α-import protein nuclear import receptor^50^, and signals through PRL1 to cell wall changes to alter gene expression and sugar-responsive metabolism^51^. However, L-Epicatechin belongs to the flavanol group of flavonoids, often in the form of sugar compounds (glycosides)^52^. Studies have shown that C-glycosylated flavonoid synthesis requires enzymes encoding flavonoid synthase, C-glycosyltransferase, glucose oxidase, rhamnosyltransferase, and glutathione S-transferase for transport to the vacuole^53,54^, suggesting the possibility that PRL1 may regulate L-Epicatechin biosynthesis by regulating or transferring glycosides in L-Epicatechin. Li et al. investigated *Tetrastigma hemsleyanum*. and found that the expression of p38/PMK-1 was associated with the synthesis of flavonoid components, which in turn ameliorated inflammation-induced damage^55^, thus it appears that the PMK gene encodes proteins that regulate the synthesis of flavonoids. PAR catalyses the reduction of 2-phenylethylamine to produce 2-phenylethanol, which is a component of the floral scent in petals^56^. Zhang et al.^57^ found that phenylalanine (Phe) of the mangiferic acid pathway is a precursor of flavonoids in *Camellia sinensis*. In addition to this, another pathway for phenylalanine is through phenylalanine metabolism catalysed by PAR to produce 2-phenylethanol, When PAR-related genes are up-regulated, the pathway is enhanced and competes with the pathway for flavonoid synthesis for phenylalanine, which inhibits flavonoid synthesis, so PAR may have an indirect effect on flavonoid synthesis.

The genes related to Kaempferol are *evm.TU.Chr14.669*, *evm.TU.Chr2.1357* and the encoded proteins are Amino acid transporter (ANT1), Sugar transport protein 8 (STP8). Ant1 and Ant2 determine anthocyanin biosynthesis and activate the synthesis of anthocyanins by affecting the expression of flavonoid 3’hydroxylase gene (*F3’H*) and *Ans* structural genes^58^. *F3’H* is a key enzyme gene for the synthesis of various other flavonoids from the flavonoid precursor dihydroflavonoids^59^, and F3’H increased the concentration of Kaempferol in *Saccharomyces cerevisiae* yeast^60^, suggesting that ANT1 may regulate the biosynthesis of Kaempferol by interacting with the structural gene *F3’H* in turn. A related study demonstrated that the transcriptome-analysed differential gene *Ans* regulates the flavonoid synthesis pathway^61^, so ANT1 may regulate flavonoid biosynthesis by acting with the Ans structural gene. STP8 may be acquired through active indirect uptake of hexoses via sugar/hydrogen co-genation, catalyses the high-affinity proton-dependent uptake of glucose and the acquisition of galactose and mannose, and is a homodimeric carrier of hexoses that is strongly expressed in plant reproductive organs. Their protein products may contribute to the uptake of sugars into pollen tubes and embryo sacs^62^. STP8 may modulate Kaempferol synthesis by regulating transport sugars.

In this study, the highest flavonoid content was found in the leaves of *Xanthoceras sorbifolia* plants from Gansu. Since previous studies have shown that environmental factors can regulate the expression of flavonoid-related genes, it was hypothesized that the suitable temperature and altitude, sufficient precipitation and light in Gansu promoted the expression of flavonoid compounds in *Xanthoceras sorbifolia evm.TU.Chr12.585*, *evm.TU.Chr2.1108*, *evm.TU.Chr9.385*, *evm.TU.Chr9.420*, *evm.TU.Chr14.669*, *evm.TU. Chr2.1357* were expressed, which promoted the process of flavonoid synthesis, and consequently increased the content of flavonoids within the leaves of *Xanthoceras sorbifolia*.

## Electronic supplementary material

Below is the link to the electronic supplementary material.

Supplementary Material 1

Supplementary Material 2

## Data Availability

The datasets generated and/or analysed during the current study are available in the National Center for Biotechnology Information (NCBI) repository, https://www.ncbi.nlm.nih.gov/sra/?term=PRJNA1224959 and https://www.ncbi.nlm.nih.gov/sra/?term=PRJNA905673.
