# Supplementary material for: Genome-wide association analysis and gene mining of flavonoids in Xanthoceras sorbifolia
Source: Sci Rep. 2025 Jul 1;15:20808. doi: 10.1038/s41598-025-00514-4 (PMC12216634; doi:10.1038/s41598-025-00514-4)
Supplement: Supplementary file 1 — Supplementary Material 1 [file 41598_2025_514_MOESM1_ESM.pdf]

Genome-wide association analysis and gene mining of flavonoids in *Xanthoceras sorbifolia*

Yuxue Huo [1], Lei Wang [1], Lu Lu [1], Dan Wu [1], Li Liu [1,2], Xiaoman Xie [1,*], and Yongjun Zhao [1,*]

[1] Key Laboratory of National Forestry and Grassland Administration on Conservation and Utilization of Warm Temperate Zone Forest and Grass Germplasm Resources, Shandong Provincial Center of Forest and Grass Germplasm Resources, Jinan 250102, China;
[2] State Key Laboratory of Tree Genetics and Breeding, National Engineering Research Center of Tree Breeding and Ecological Restoration, Key Laboratory of Genetics and Breeding in Forest Trees and Ornamental Plants, Ministry of Education, College of Biological Sciences and Biotechnology, Beijing Forestry University, Beijing 100083, China;
* Correspondence: xxm529@126.com, Tel.: +86-13969151903(X.X.); 13305317857@163.com, Tel.: +86-13305317857(Y.Z.)

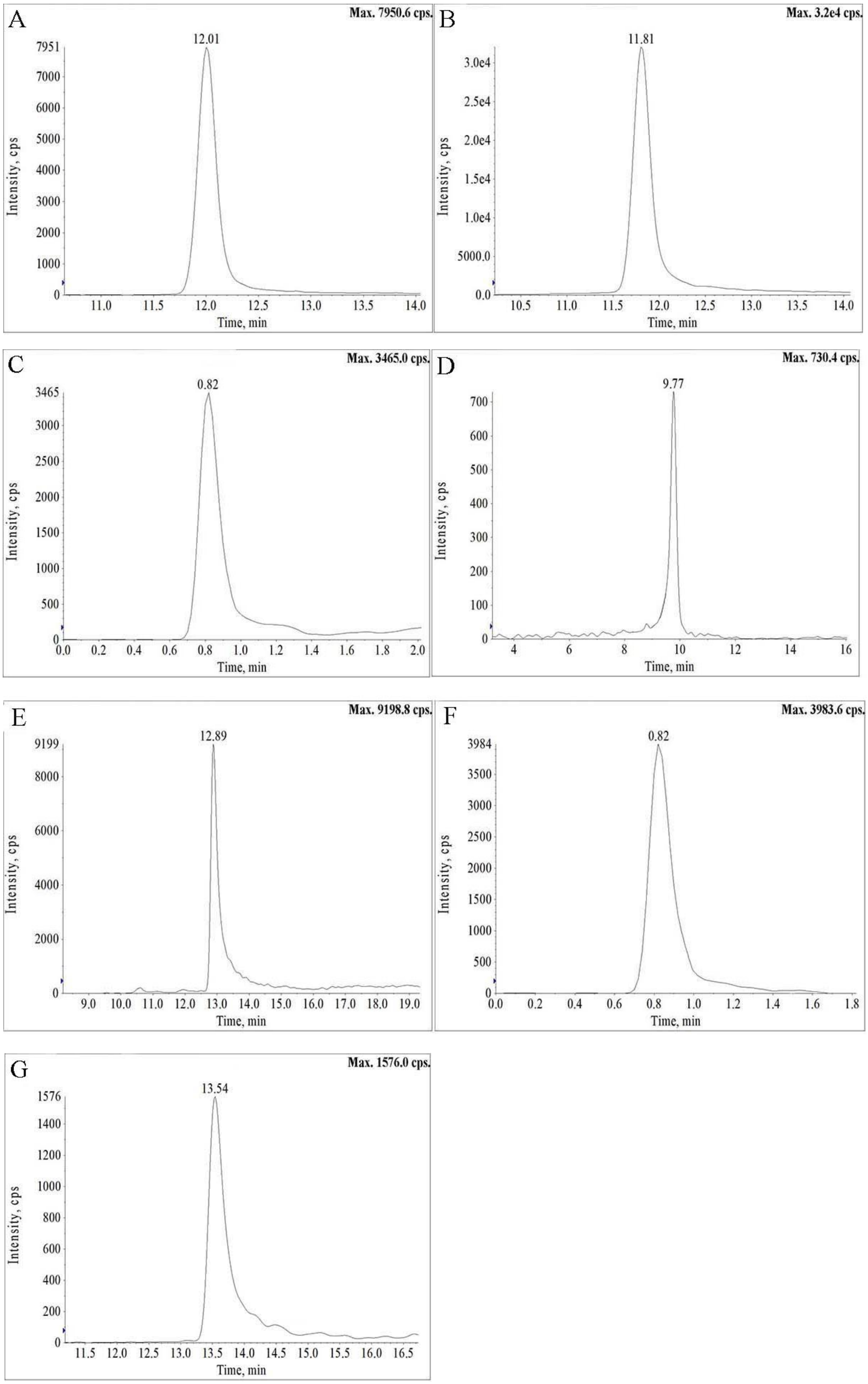

Fig. S1 XIC diagram of 7 flavonoid compound standard (A) Rutin standard; (B) Myricitrin standard; (C) (-)-Epigallocatechin standard; (D) L-Epicatechin standard; (E) Quercetin standard; (F) (+)-Gallocatechin standard; (G) Kaempferol standard.

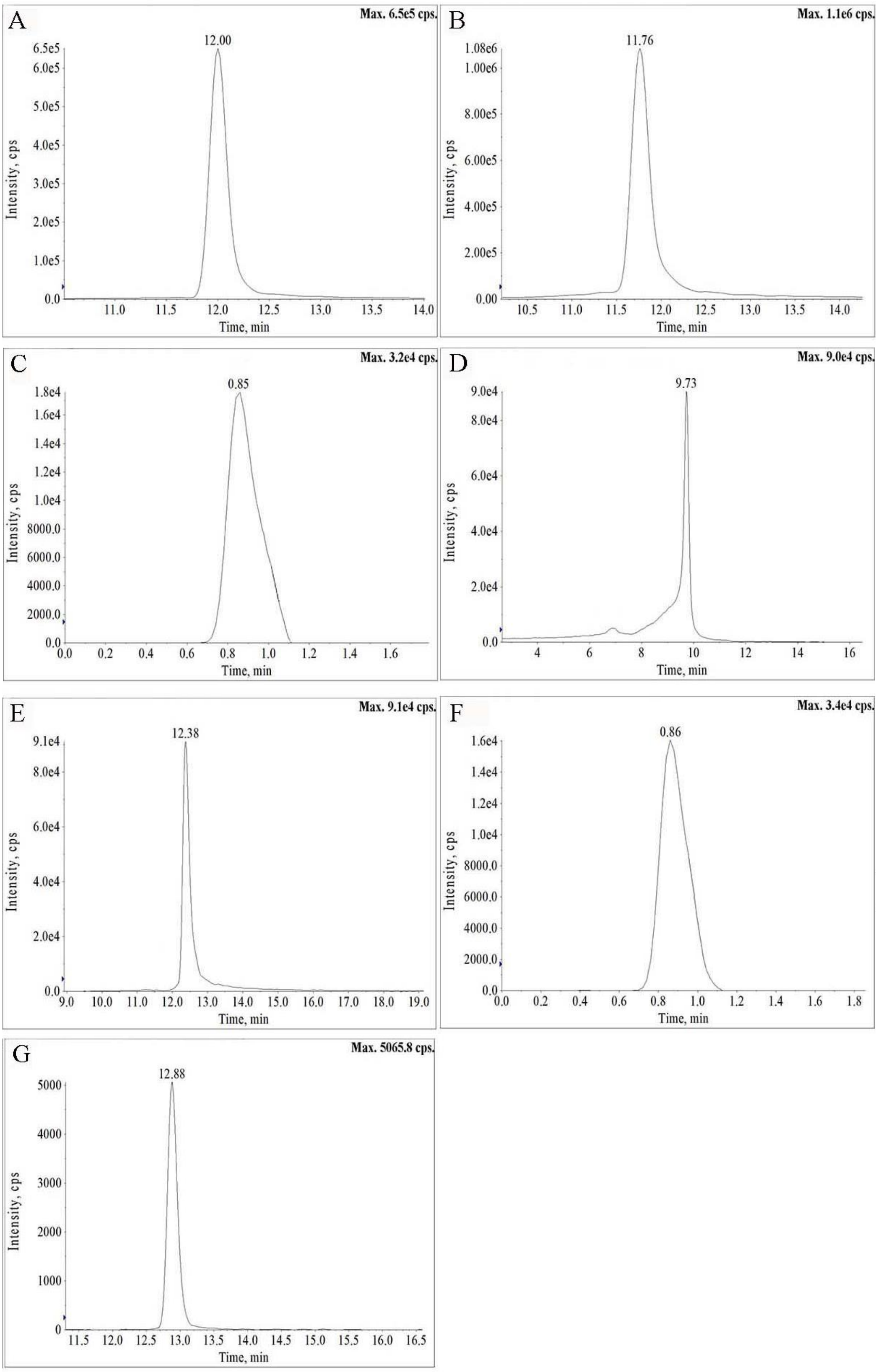

Fig. S2 XIC diagram of 7 flavonoid compound samples (A) Rutin sample; (B) Myricitrin sample; (C) (-)-Epigallocatechin sample; (D) L-Epicatechin sample; (E) Quercetin sample; (F) (+)-Gallocatechin sample; (G) Kaempferol sample.

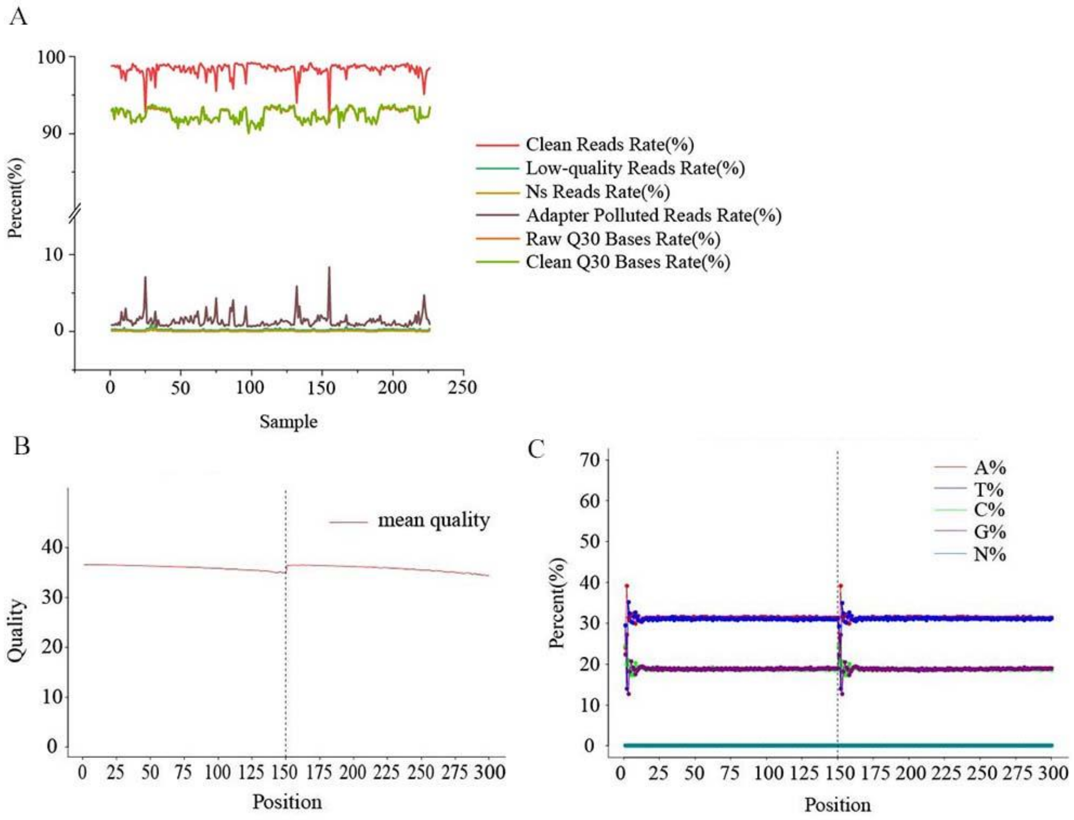

Fig. S3 (A) Statistical analysis of data filtering; (B) Mass value distribution diagram of sample 2021WD035; (C) Base distribution diagram of sample 2021WD035

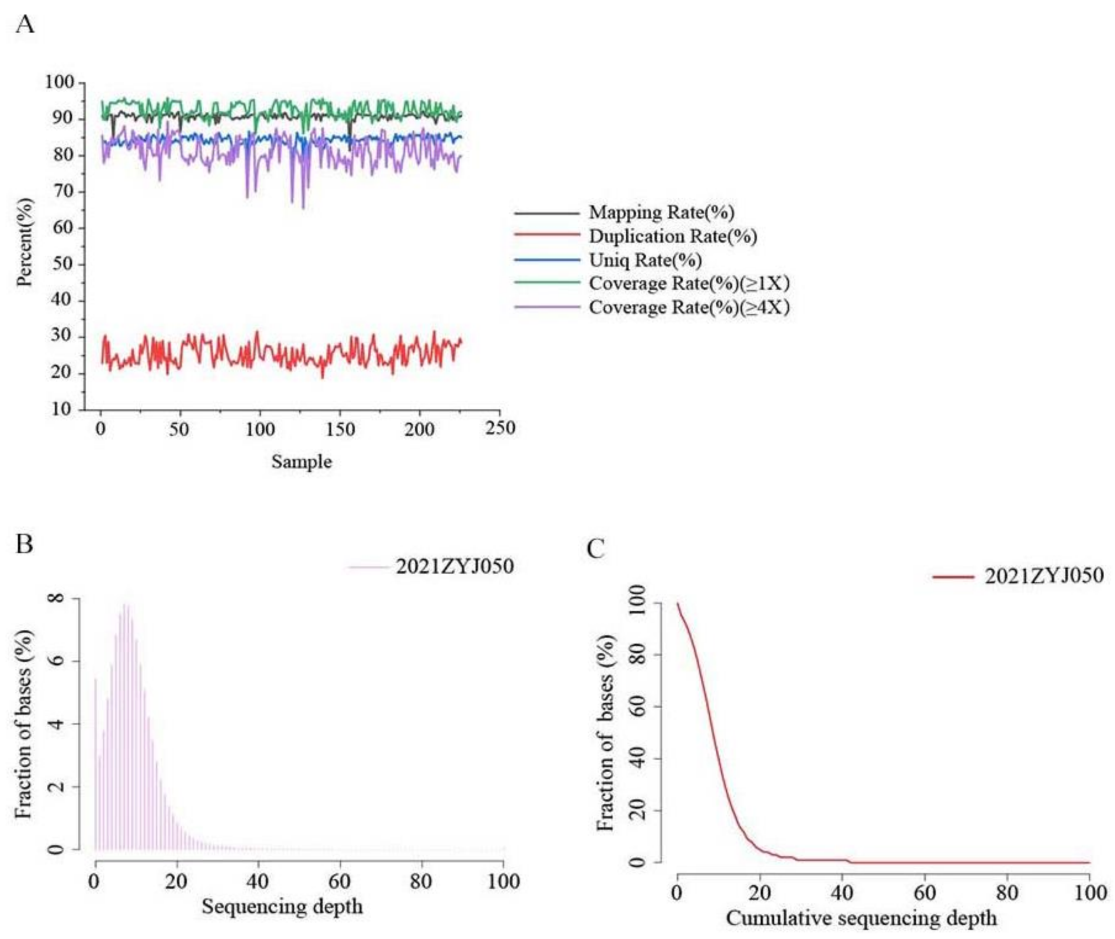

Fig. S4 (A) Comparison data statistics; (B) Single base depth distribution map of sample 2021ZYJ050; (C) Cumulative depth distribution map of sample 2021ZYJ050

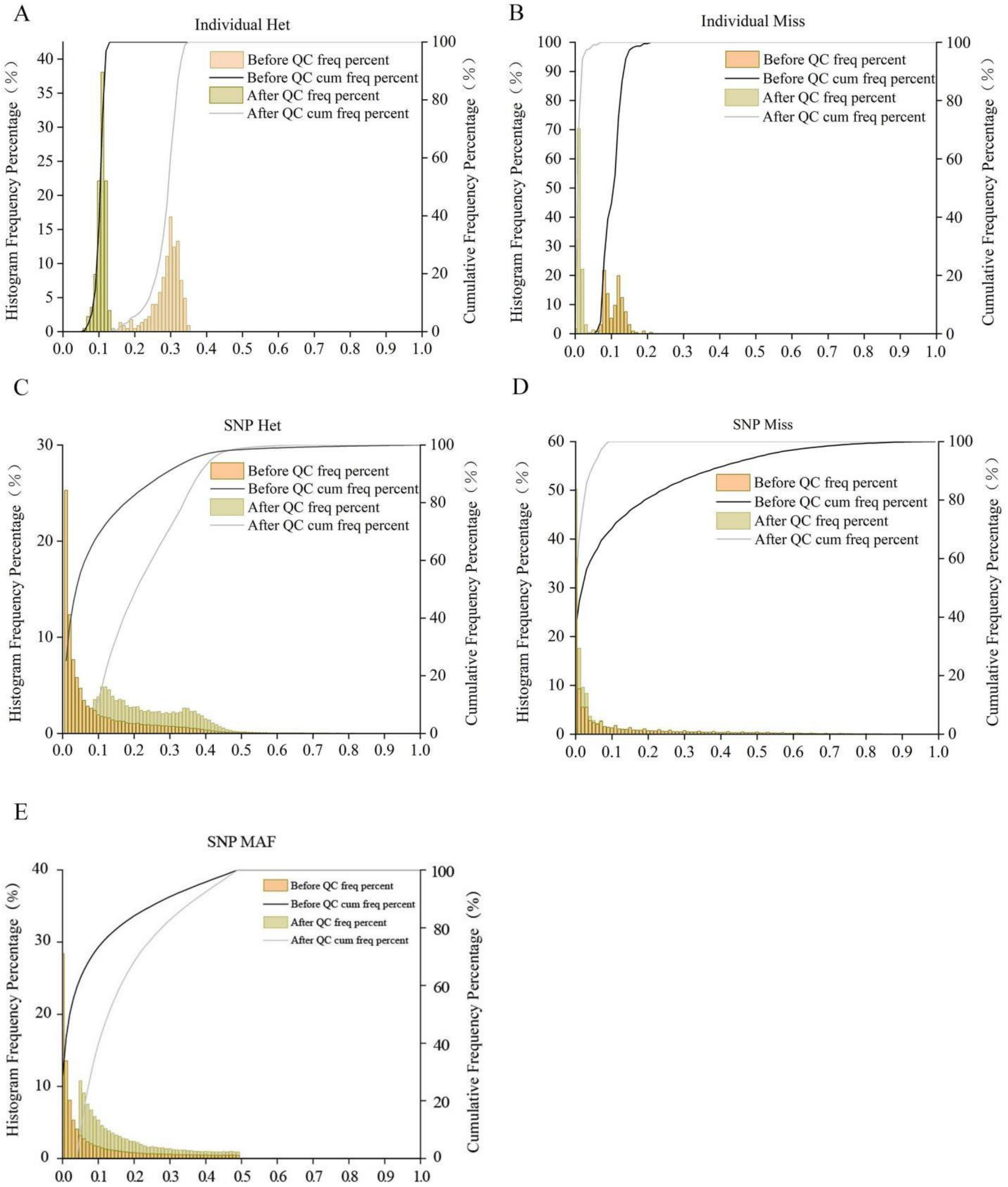

Fig. S5 Statistical chart of comparison before and after SNP quality control (A) Statistical chart of individual heterozygosity before and after quality control; (B) Statistical chart of individual missing rate before and after quality control; (C) Statistical chart of SNP site heterozygosity before and after quality control; (D) Statistical chart of SNP deletion rate before and after quality control; (E) Statistical chart of SNP minimum allele frequency before and after quality control
